# Turning Semicircular Canal Function on Its Head: Dinosaurs and a Novel Vestibular Analysis

**DOI:** 10.1371/journal.pone.0058517

**Published:** 2013-03-13

**Authors:** Justin A. Georgi, Justin S. Sipla, Catherine A. Forster

**Affiliations:** 1 Department of Anatomy, Arizona College of Osteopathic Medicine, Midwestern University, Glendale, Arizona, United States of America; 2 Department of Anatomy and Cell Biology, University of Iowa, Iowa City, Iowa, United States of America; 3 Department of Biological Sciences, The George Washington University, Washington D.C., United States of America; Royal Ontario Museum, Canada

## Abstract

Previous investigations have correlated vestibular function to locomotion in vertebrates by scaling semicircular duct radius of curvature to body mass. However, this method fails to discriminate bipedal from quadrupedal non-avian dinosaurs. Because they exhibit a broad range of relative head sizes, we use dinosaurs to test the hypothesis that semicircular ducts scale more closely with head size. Comparing the area enclosed by each semicircular canal to estimated body mass and to two different measures of head size, skull length and estimated head mass, reveals significant patterns that corroborate a connection between physical parameters of the head and semicircular canal morphology. Head mass more strongly correlates with anterior semicircular canal size than does body mass and statistically separates bipedal from quadrupedal taxa, with bipeds exhibiting relatively larger canals. This morphologic dichotomy likely reflects adaptations of the vestibular system to stability demands associated with terrestrial locomotion on two, versus four, feet. This new method has implications for reinterpreting previous studies and informing future studies on the connection between locomotion type and vestibular function.

## Introduction

Three semicircular canals are present in the neurocranium of all vertebrates. The fluid-filled ducts within these canals sense angular accelerations of the head and operate in conjunction with reflex arcs to cervical and extraoccular muscles to stabilize the head and maintain visual fixation. As such, functional morphologists have sought to correlate duct size and shape with motions experienced by the body, particularly those encountered during locomotion. Although hypotheses linking the morphology of the semicircular canals to locomotor type go back to the first surveys of the vestibular system [1]–[3], it was not until 1963 that Jones and Spells [4] made the first comprehensive attempt to quantify this relationship. This pioneering study assumed two possible scenarios of similarity in duct function relative to body proportions: geometrical similarity (all vertebrate heads are roughly the same mass relative to body mass), and dynamic similarity (all vertebrate heads produce the same stress on the neck and body relative to body mass) [4]. By reducing these assumptions to allometric equations, they predicted that the radius of curvature of the semicircular ducts should have an allometric scaling exponent between 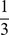 and 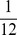 with respect to body mass [4].

Jones and Spells tested their predictions by analyzing semicircular duct measurements in mammals, birds, reptiles, and fishes [4] of known or estimated body mass, and interpreted their results as an indication that semicircular duct response is adaptively correlated to body mass [4]. This conclusion has formed the theoretical basis for numerous subsequent studies [5]–[13]. By accepting Jones and Spells' assumptions, these studies tend to interpret residual variation from the semicircular duct size vs. body mass line of allometry as being a strong measure of adaptive duct function. For example, in their broad comparison of primates to other mammals, Spoor *et al.* found that specimens subjectively classified as fast or agile tended to have semicircular canals with radii of curvature larger than expected for their body size, whereas specimens classified as slow tended to have smaller canals [6]. Although they reported statistical support for their findings, there was marked overlap between their agility categories, and some specimens (*e.g.*, *Ateles geoffroyi*) have canals that are not sized-matched with their assigned agility [6]. Such studies have demonstrated the utility of the vestibular system to investigations of vertebrate locomotion, while simultaneously suggesting that duct response may not correlate most accurately with body mass.

Jones and Spells' two assumptions depend on the assertion that the mass of a vertebrate's head is consistently proportional to body mass [4], reductively equating the moving object containing the vestibular system (the head) to the whole body. However, if there is no simple or consistent correlation between head and body mass, these assumptions will not accurately describe the physical system to which the semicircular ducts are adapted. The semicircular ducts are stimulated by motions of the head; they transduce angular motion in order to modify the position of the head, body, and eyes [14]. Whereas most vestibular stimuli undergo higher-level neural processing prior to somatic actualization, near-instantaneous head and eye adjustments are accomplished through high speed reflex arcs (vestibulo-ocular and vestibulocollic) which are less modulated by other neuronal inputs [15]. Input from the semicircular ducts acts to effectively isolate the head with respect to the body's motion. We argue that it is these reflexes, crucial for continuous maintenance of head position and visual stabilization, which drive adaptations of the semicircular ducts. As such, head mass may provide a better functional correlate for duct size than does body mass.

The physical property that is most influential on the movement of the head in response to an applied torque is the moment of inertia, an object's inherent resistance to changes of angular momentum. Moment of inertia, however, is an impractical measurement to take in a biological context for the primary reason that it represents the distribution of mass relative to the rotation's axis, which is not likely to be constant in either position or orientation for a moving animal. No single metric is expected to be a perfect proxy for moment of inertia and there are theoretical and practical justifications for many different options. In this study, two separate metrics are used to represent the moment of inertia of the head, skull length and head mass.

The mass of the head has the stronger theoretical justification of the two head metrics considered in this study. It directly represents one of the factors in the calculation of moment of inertia, the sum of the masses of all the individual segments in the object. It is also the portion of the moment of inertia that is independent of the position and orientation of the axis of rotation, in contrast to the distance of each point from the axis of rotation. This independence means head mass is expected to correlate equally well with each canal for which there is a functional relationship. Furthermore, as this study seeks to examine the justification of assumptions behind Jones and Spells' use of body mass to study semicircular duct allometery, this metric is appropriate as it is invoked directly and indirectly in their theoretical considerations [4]. The difficulty with using head mass to represent the moment of interia is that it is not a commonly reported metric in the literature for modern animals and even less so for extinct animals. As a result, the mass of the head must be estimated and this may reduce the reliability of this metric.

The length of the skull is an advantageous metric from a practical standpoint. This property is widely reported in the literature for many types of animals both modern and fossil. Often it is a direct measurement from a specimen and, therefore, more reliably represents the animal in question than any estimated parameter. In addition, as a result of these practical considerations, this is a metric already used widely in studies of head size across a broad array of vertebrates which may facilitate correlation of semicircular duct studies with a broader body of comparative work. Length of the skull, however, is expected to be a less robust proxy for moment of inertia than head mass and therefore may not provide the same breadth of functional significance. When considered with respect to moment of inertia, the length of the skull can represent the distance to the axis of rotation. However, it only represents that distance with regards to mass segments out near the anterior tip of the skull and then only if the axis of rotation passes near the occipital condyle rather than any other portion of the body axis and that rotation is perpendicular to the midline axis of the skull. Of the rotational axes that are most functionally significant with respect to each semicircular duct in isolation, two (the axes for the anterior and posterior ducts) are obliquely oriented with respect to the body midline; only the axis of rotation most closely associated with the function of the lateral duct is approximately perpendicular to the body midline. Thus, in contrast to the mass of the head, the length of the skull is expected to have a much stronger correlation with the lateral semicircular duct than with the two vertical ducts even if a functional relationship exists for all three.

**Figure 1:**
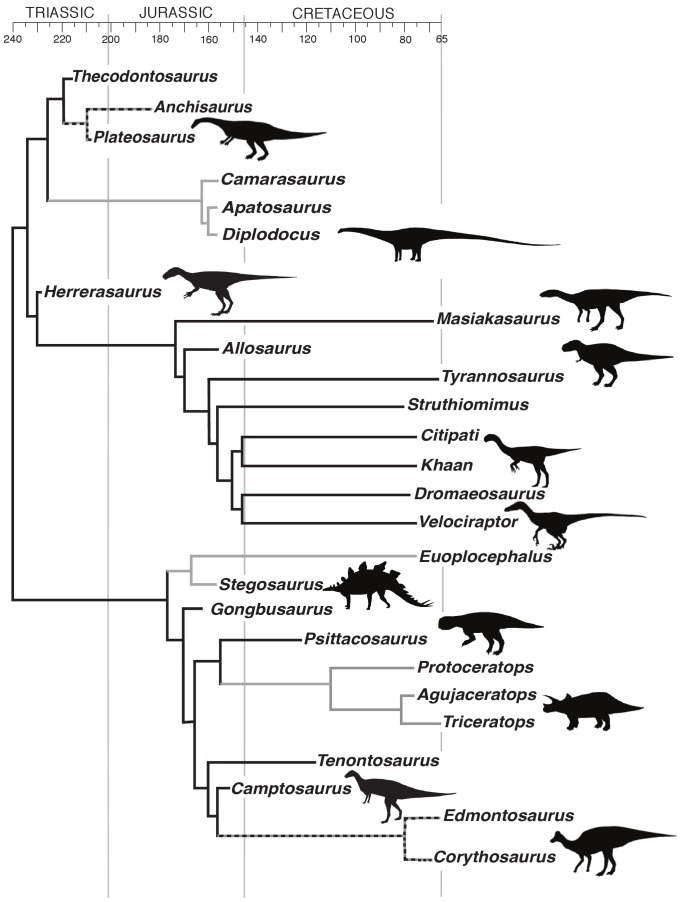
Specimen phylogeny. Calibrated phylogeny of the twenty-six non-avian dinosaur taxa examined in this study [26]–[29]. Head size relative to body size varies broadly across dinosaurs with extremes ranging from the small-headed sauropods to the large-headed ceratopsids. Black lines: bipeds, gray lines: quadrupeds, and dashed lines: contentious primary locomotor types.

In non-avian dinosaurs (henceforth referred to as dinosaurs), any assumption of head size similarity across body mass is critically flawed (Figure 1). Two notable examples are the extreme small relative head size in sauropods, such as *Apatosaurus* [16], and extreme large relative head size in ceratopsids, such as *Triceratops* [17]. These two extremes not only differ from most other dinosaurs, but also from most extant vertebrates [16], [17]. Dinosaurs also exhibit functionally distinct locomotor postures, bipedalism and quadrupedalism. Bipeds must spend a large percentage of the step cycle with their center of gravity outside their very small area of support (a single foot in contact with the ground), producing a constant tendency to fall. In contrast, quadrupeds typically have an area of support that is defined by two or more feet (depending on gait and speed) and, thus, have a greater ability to keep the center of mass over that enlarged area of support. With a broad array of relative head sizes and two modes of locomotion imposing different balance requirements, dinosaurs offer a unique model system to test whether functional adaptation of the semicircular duct system is more tightly correlated with either head size metric or body mass.

## Materials and Methods

Semicircular canal morphology data were collected from X-ray computed tomography (CT) scans of 29 dinosaur specimens representing most of the major clades within dinosaurs (Table S1). Data from congeneric specimens were averaged resulting in a dataset of 26 different genera (Figure 1). Dinosaur taxa were divided into two primary functional groups based on the typical limb number employed during locomotion: bipedal (n = 14) and quadrupedal (n = 8). Four taxa for which the skeletal, functional, and ichnofossil data are ambiguous (*i.e.*, stance is unknown, or both locomotor modes were likely utilized) were left uncategorized. Specimen sampling was sufficient such that both of the functional groupings contained representatives from the two major clades of dinosaurs, Saurischia and Ornithischia, and in most cases there are bipeds and quadrupeds representing the same subgroups. To achieve the most robust functional comparisons, only primarily terrestrial dinosaurs were used; volant and secondarily terrestrial dinosaurs (flying and secondarily flightless birds) were excluded to control for any effect of neural integration with locomotion that might arise when an organism transitions into a completely different mode of locomotion (flying) and back again.

**Figure 2:**
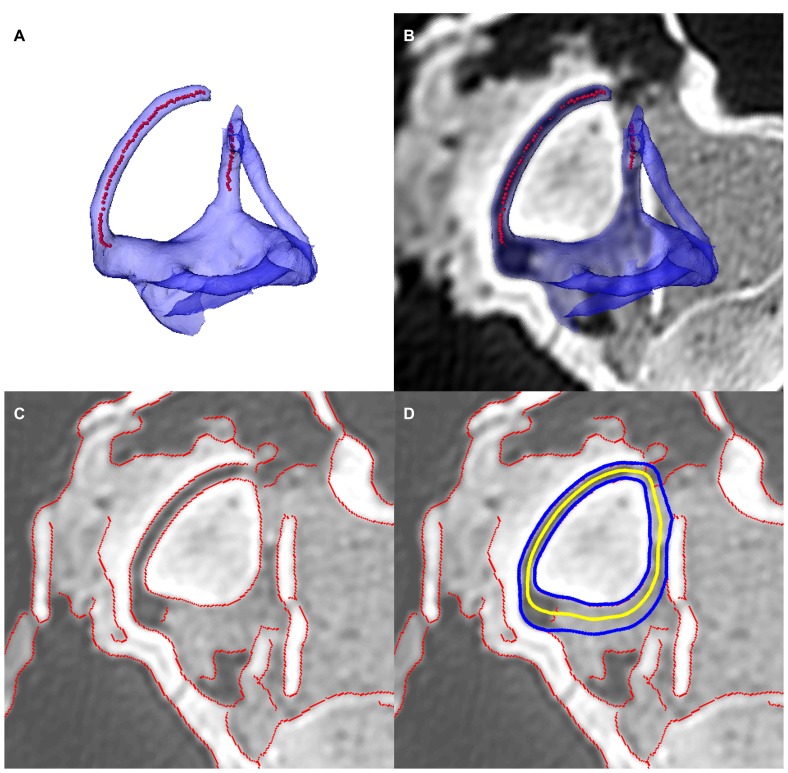
Canal midline estimation method. (A) Multiple points, each representing the centroid of a section through the slender portion of the canal, are manually digitized. (B) A best fit plane through these points is calculated using principal components. (C) Parameters of a canny edge-finding algorithm are manually adjusted to ensure maximal automatic edge reconstruction on the best fit plane image. Any missing sections of the canal edges are reconstructed using linear interpolation for small sections and replication of the complimentary edge for larger sections. (D) An average midline path between the internal and external canal edges is calculated to represent the course of the membranous duct. The area enclosed by this average path is used as the canal size metric in this study.

Using Amira 5.2 [18], points were manually placed at the centroid of every identifiable semicircular canal lumen section in the CT scans. Canal planes were defined as the best-fit plane by principle components reorientation of these points, and planar images of the canals were extracted. The planar semicircular canal images were processed in Matlab R2010a [19]. External and internal canal walls were outlined using a canny edge-finding routine. Small gaps in the calculated walls were filled with linear interpolation while larger gaps were filled by replication of the complimentary section of the corresponding wall. The completed wall outlines were then averaged to produce an estimated midline closed-circuit through the semicircular canal and utricular region of the vestibule (Figure 2). Semicircular duct function was then represented in this analysis by calculating the area enclosed by this estimated planar canal circuit.

This method produces planar area measurements analogous to other, more computationally intensive methods (*e.g.*, Gunz et al., [20]); the procedure used, however, is more compatible with fossil specimens, being suitable for use both with damaged canals and with labyrinths that have been filled with sediment during fossilization. Although some part of the bony labyrinth is often preserved in fossilized braincases, it is rare that the system is preserved in its entirety. Methods that require a full 3-dimensional reconstruction of the bony labyrinth [20], [21] significantly restrict the specimens that can be analyzed. Similarly, methods that rely on a single greyscale threshold to identify the boundaries of the bony labyrinth or produce a reconstruction [20] are less suitable for fossils where heterogeneous matrix in-fills the labyrinth or, as is commonly the case, where matrix filling is only partial. In these instances, our canny edge-detection based approach (*i.e.*, gradient identification independent of absolute value) provides improved reconstruction over a threshold based method.

Body mass estimates were collected from the literature. Although there is no significant or systematic difference between limb bone measurement mass estimation and polynomial mass estimation methods [22], wherever possible (21 of 29 specimens), preference was given to body masses estimated using Seebacher's polynomial estimation approach [22] due to the range of available data. In four cases where no body mass estimate was found in the literature (Table S2), body masses were estimated using limb bone allometric equations [23].

The length of the skull for each taxon was collected from literature values. In most cases, the value used is the basicranial length. The two exceptions to this are *Triceratops* and *Agujaceratops* which have significant portions of the skull extending posteriorly beyond the occipital condyle. For these two specimens the length of the skull includes the frill [17].

To estimate head mass, the total length of the animal was taken from literature values, reconstructions, and original measurements, and the body mass estimates were scaled to the ratio of skull length to body length.

In order to preserve equivalence of units, the square root of the area enclosed by the semicircular canal was compared against the cube root of each mass estimate (body or head) and untransformed skull length. The comparisons were performed in log-log space in order to be able to represent the expected allometric relationships by fitting linear regressions using a Standardized Major Axis (SMA) method. Where correlations with slopes significantly different from zero (p<0.05) were obtained, the regression lines of the two locomotor groups were compared for statistical similarity of slope and elevation using the Smatr package version 2.1 [24] for R statistical computing software version 2.10.1 [25].

Phylogenetic Independent Contrast (PICS) tests for phylogenetically based autocorrelation within the data were run using the phylogeny in Figure 1, which was constructed from unpublished data (Gongbusaurus) and recent literature [26]–[36]. Using the PDAP package version 1.15 [37] for Mesquite version 2.74 [38], Pearson product-moment correlations were computed for both bipeds and quadrupeds. In order to minimize any potential effects of having numerous large gaps in the topology of the tree, two different models of branch length were used in the PICS analyses and their results compared for consistency: 1) branch lengths were set to the natural log of calculated minimum divergence times based on calibrated ages of known nodes [30]–[36], and 2) branch lengths were set to 1.

## Results

**Figure 3:**
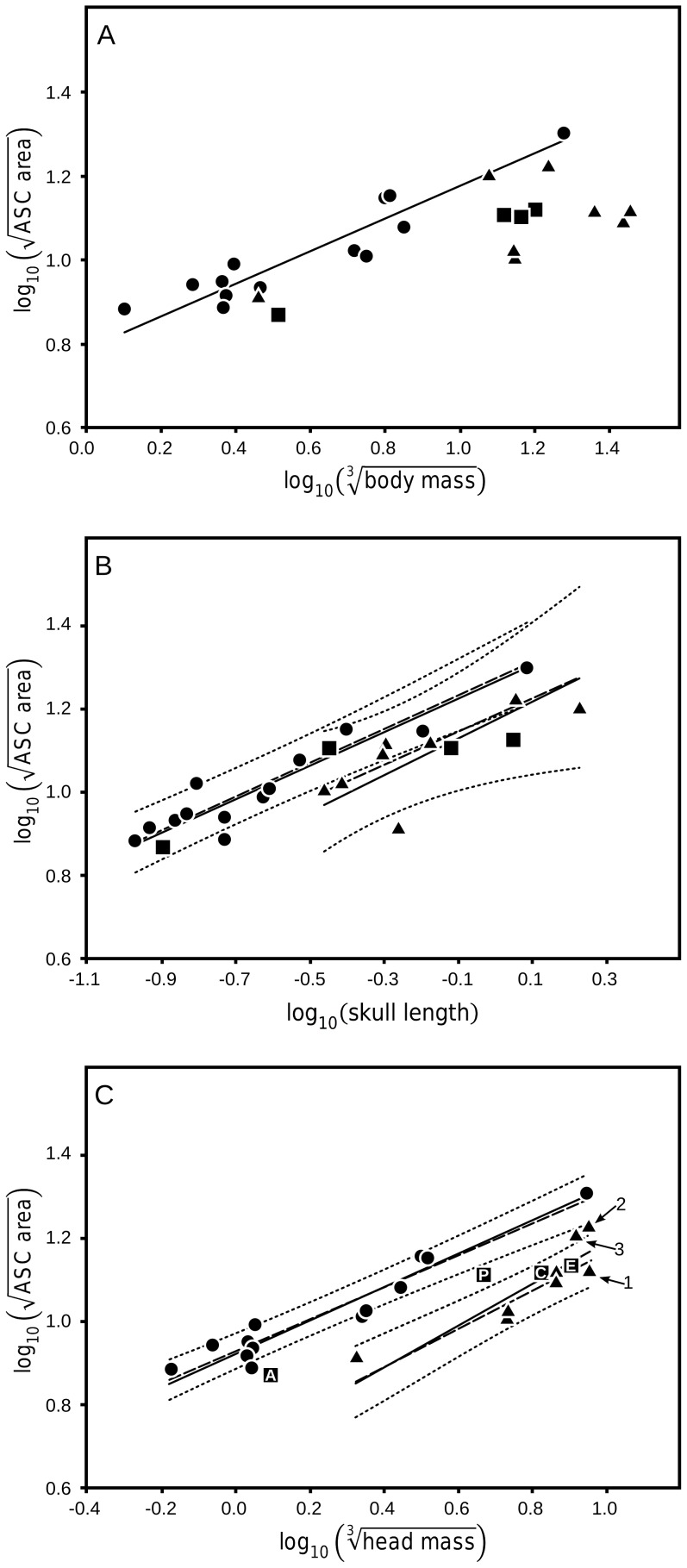
Anterior semicircular canal size in dinosaurs. Anterior semicircular canal (ASC) area enclosed versus (A) body mass, (B) skull length, and (C) head mass. The bipedal correlations in (A), (B), & (C) are significant and the regressions are similar in slope. Quadrupedal taxa in (A) exhibit no significant correlation in contrast to (B) and (C) where the quadrupedal correlations are significant. In (B) and (C) the slope of the quadrupedal regression is statistically similar to the bipedal slope but significantly separated from it by a decrease in intercept. The intercept difference between the bipedal and quadrupedal groups in (C) is greater in magnitude than in (B) and when combined with the much stronger correlations in (C) this comparison produces the most robust discrimination between bipedal and quadrupedal taxa. The simplicity of the head mass estimation method employed in this study results in several notable estimation errors: the head size in *Apatasaurus* (1) is over-estimated and the head sizes of the derived ceratopsians, *Triceratops* (2) and *Agujaceratops* (3) are under-estimated. In each case, more accurate head mass estimations would shift these taxa closer to the quadrupedal regression line. Solid lines: standardized major axis regressions for significant correlations, dashed lines: phylogenetically correct ordinary least square regression (PCOLS), and dotted lines: 95% confidence interval for each group around the PCOLS. •: bipeds, ▴: quadrupeds, and ▪: taxa with ambiguous posture (A: *Anchisaurus*, C: *Corythosaurus*, E: *Edmontosaurus*, and P: *Plateosurus*).

Table 1 shows the correlations between each of the three canal measurements and body mass, skull length, and head mass. In bipedal dinosaurs the anterior semicircular canal (ASC) area exhibits a strong correlation with body mass; in contrast, the ASC area of quadrupedal dinosaurs has no significant correlation with body mass (Figure 3a). However, when ASC area is compared against skull length (Figure 3b), there are four noticeable changes: 1) the correlation within bipeds improves, 2) a strong and significant correlation appears within quadrupeds, 3) the slopes of the bipedal (0.178) and quadrupedal (0.195) regressions become statistically similar (difference p = 0.763), and 4) there is a significant elevation difference (the bipedal regression is significantly higher) between the two groups (p = 0.003). When compared against estimated head mass (Figure 3c), this pattern of changes becomes more pronounced. The correlations for both bipeds and quadrupeds are stronger than with either body mass or skull length. As before, the slopes of the bipedal (0.396) and quadrupedal (0.501) regressions are statistically similar (difference p = 0.285). There is a significant elevation difference (again, the bipedal regression is higher) between the two groups (p<0.001), but where there was substantial overlap in the 95% confidence areas of the two groups when using skull length as the comparison factor, with estimated head mass, there is almost no overlap.

**Table 1:** Pearson correlation coefficients between semicircular canal area and mass estimates and skull length for bipedal and quadrupedal dinosaurs.

		ASC	PSC	LSC
Bipedal	Head mass	0.962*	0.955*	0.826*
	Skull length	0.942*	0.911*	0.866*
	Body mass	0.932*	0.947*	0.788*
	n	13	10	12
Quadrupedal	Head mass	0.888*	0.674	0.482
	Skull length	0.751*	0.928*	0.771*
	Body mass	0.613	−0.301	0.170
	n	8	7	7

In all cases except the skull length correlation with the PSC in bipedal dinosaurs, both the head size correlations represent increases in the correlation coefficient above that for the body mass estimates. The increases in correlation coefficient for the quadrupedal taxa are substantially larger than those of the bipedal taxa. With the ASC data, this increase in the quadrupedal correlation includes changing from a non-significant correlation with body mass to a significant correlation with both head size metrics. The PSC-head mass correlation is nearly significant (critical value of 0.754). Between the head size metrics, head mass produces stronger correlations than skull length in the vertical canals except for the quadrupedal PSC correlations (see discussion). In contrast, however, the LSC correlations for both bipedal and quadrupedal groups are stronger with the skull length than head mass, suggesting that skull length is more functionally significant for the LSC than either of the vertical canals. ASC: anterior semicircular canal, PSC: posterior semicircular canal, and LSC: lateral semicircular canal. *- indicates a significant correlation at the p = 0.05 level.

When the area enclosed by either the posterior or lateral semicircular canals (PSC or LSC, respectively) is compared to body mass, the same pattern emerges: bipeds show a moderately strong correlation with each metric while quadrupeds exhibit no significant correlation. When PSC and LSC area are compared to head mass, the correlations within the bipedal dinosaur data improve. In contrast to what was observed with the ASC, the areas enclosed by the PSC and LSC do not correlate significantly with head mass estimates in quadrupedal dinosaurs.

**Figure 4:**
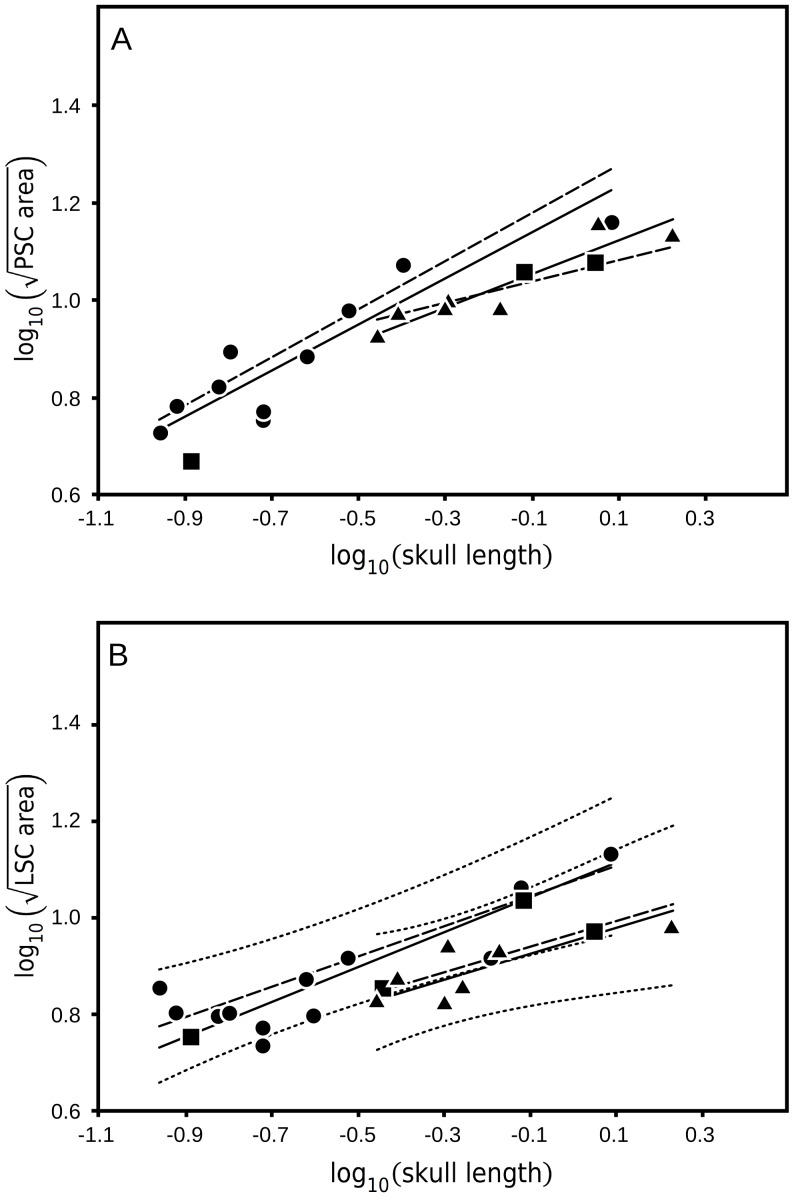
Posterior and lateral semicircular canal size with respect to skull length. (A) Posterior semicircular canal (PSC) area enclosed versus skull length, and (B) Lateral semicircular canal (LSC) area enclose versus skull length. Skull length significantly correlates with both PSC and LSC area enclosed in both bipedal and quadrupedal dinosaurs. The PSC regressions of the two groups are similar in slope and regression intercept, indicating that skull length cannot be used with PSC size to distinguish bipeds from quadrupeds. The LSC regressions of the two groups also share a common slope, but are significantly separated by regression intercept with the bipeds elevated above the quadrupeds. This separation is similar to the anterior semicircular canal (ASC) size comparison with skull length and much less robust than the ASC size comparison with estimated head mass (Figure 3). Solid lines: standardized major axis regressions for significant correlations, dashed lines: phylogenetically correct ordinary least square regression (PCOLS), and dotted lines: 95% confidence interval for each group around the PCOLS. •: bipeds, ▴: quadrupeds, and ▪: taxa with ambiguous posture.

Skull length comparisons to the PSC and LSC (Figure 4, a and b), however, result in correlation patterns that are slightly different from each other and from the pattern observed in the estimated head mass correlations. In bipedal dinosaurs, skull length correlates more weakly with area enclosed by the PSC than does either body mass or head mass. This is the only instance in any of the comparisons where one of the head parameters has a lower magnitude correlation than body mass. In contrast to this decrease, the quadrupedal correlation between PSC and skull length shows the greatest increase in magnitude over the body mass correlation of any of the comparisons. As with the ASC comparison, this increased correlation is significant where the body mass correlation was not. A comparison of significant bipedal and quadrupedal correlations shows that the slopes are statistically similar (biped: 0.204, quadruped: 0.151, difference p = 0.205), but there is no significant separation between the two regressions (difference p = 0.159).

When compared to LSC area, skull length produces a pattern of correlations unlike any of the other correlations. In this case, both the bipedal and quadrupedal correlations with skull length are stronger than with head mass. As was the case with the skull length and PSC correlation, this correlation shows a dramatic increase in magnitude. Like the PSC correlation with skull length, the slopes of the bipedal (0.157) and quadrupedal (0.119) groups are statistically similar. Unlike with the PSC, however, skull length compared to LSC area statistically separates bipeds from quadrupeds (difference p = 0.002).

Four of the nine comparisons produced statistically significant regressions for both bipedal and quadrupedal groups. Of these four, three (ASC vs head mass, ASC vs skull length, and LSC vs skull length) also demonstrated statistical differentiation between the two groups based on the regression intercept (Figures 3 and 4), with the bipedal regression having the higher intercept in all three cases. The strongest separation between the two groups (both statistically and visually) is observed in the ASC vs estimated head mass. As a result, this comparison provides the most clarity when examining the taxa unassigned to a locomotor group.

Two different patterns are evident in the taxa with contentious locomotion when the ASC is compared to head mass. The two sauropodomorphs, *Anchisaurus* and *Plateosaurus*, lie between the two functional groups and outside of the 95% confidence intervals for those groups. In contrast, the two hadrosaurs, *Corythosaurus* and *Edmontosaurus*, lie unambiguously within the range of the quadrupedal group.

Both branch length models produced very similar results in the PICS analyses (the greatest difference in PICS correlation coefficients between the two models is 0.101). The magnitudes of the correlations in the PICS analysis are higher than in the standard analysis for all comparisons except for the lateral canal comparisons in the bipeds and the head mass comparison to the posterior canal in the quadrupeds. Similarly, the pattern of correlation significance in the PICS analysis parallels the standard analysis with only two differences: in the PICS analysis, anterior semicircular canal comparison to body mass and the posterior semicircular canal comparison to head mass in the quadrupeds are significant where neither comparison is significant in the standard analysis. None of the significant correlations reported with the standard analysis were rendered insignificant in the PICS analysis. As with the standard analysis, in all six comparisons the magnitude of the correlation between a canal metric and head mass was higher than for body mass.

## Discussion

Estimating head mass in extinct organisms is difficult. The method employed in this study was selected because of the availability of the data in the literature, not because it is considered highly accurate or complete. We recognize it is rudimentary as evidenced by cases where the estimates do not accurately represent the actual head size of the organism. The most striking example of this is with the diplodocid sauropods, which are notable for their relatively small head size [16]; yet, with this method, *Apatosaurus* (Figure 3, ▴1, skull length = 0.6 m) has an estimated head mass larger than that of *Tyrannosaurus* (skulls up to 1.5 m long) and *Triceratops* (skulls up to 2.1 m long), a clear over-estimation.

Conversely, this method likely underestimates the mass of heads with horns or other bony ornamentation. For example, ceratopsids, such as *Triceratops* and *Agujaceratops* (Figure 3, ▴'s 2 & 3), are recognized as having some of the largest skulls of any land vertebrate in history [17]. In this analysis, the head of *Triceratops* is estimated at 701 kg, only 30 kg heavier than that of *Tyrannosaurus*. Our estimate, however, does not take into account ornamentation such as the nasal or supraorbital horns of this taxon. Henderson's estimates for the mass of the supraorbital horns in *Triceratops* [39] would add 26 kg to the mass estimate used in this study. Although adjustment of the head mass values for *Triceratops* and *Agujaceratops* by the Henderson estimates does increase the correlations of head mass with each of the canals (e.g., ASC correlation with head mass increases to 0.894 and PSC correlation with head mass increases to 0.695), it is not sufficient to change any of the significances at the p = 0.05 level.

Other, more compound, metrics for estimating head mass, such as elliptical volume, were considered. The goal of this study, however, was to maximize the number of taxa and the lack of available data for such a metric was too restrictive. With this study, we present evidence that metrics of head size, even this simple method of estimating head mass, provide considerably improved size parameters over the more typical body mass for a functional analysis of semicircular canals. As such, we have not refined or adjusted any individual calculated head mass; all of the data presented above and analyzed in the following discussion are based on our rudimentary head mass algorithm outlined above.

The overall pattern of correlation magnitude and significance shows a strong correspondence to the patterns expected based on the hypothesis that semicircular duct function scales with the moment of inertia of the head and not with body mass. In all comparisons, except for the comparison of skull length to PSC area in bipeds, the head size metrics correlate more strongly with the area enclosed by the semicircular canal than does body mass. In none of the comparisons is a significant correlation with body mass rendered insignificant when a head size parameter is used instead. Furthermore, these data demonstrate that, as hypothesized, more dimensionally restricted metrics such as skull length are more appropriate size parameters only for specific semicircular canals based on their relationship with the axis of rotation maximally associated with that canal. These facts alone are enough to suggest that vestibular functions represented by the area enclosed by the semicircular canals are adapted to head parameters rather than body mass. Additionally, examining these correlations within the functionally defined locomotor groups adds overwhelming support for this hypothesis.

For bipedal taxa, the body mass correlations are all significant. Skull length comparisons improve the strength of the correlations with both ASC and LSC. The skull length to PSC comparison, however, is the only example of a head size parameter that has a weaker correlation than body mass. This is not surprising; skull length was not expected to be the head parameter that performed better with either ASC or PSC, but instead was expected to show the strongest affinity with the LSC. Similarly, improvements gained using head mass correlations represent only minor adjustments to the data. These results are expected, as bipedal dinosaurs, despite the phylogenetic distance between them, all share a similar body form and relative head size (Figure 1). For example, within theropods, head size is geometrically similar enough that body mass can be predicted in most groups on the basis of skull length with the same accuracy as other methods [40]. Nonetheless, within these bipedal taxa, there is some variation of head size (*e.g.*, head length is 10.5% of body length in *Masiakasaurus* and 5.8% in *Struthiomimus*). When these small variations are factored into the comparison by the use of a head parameter, a stronger correlation with semicircular canal size emerges.

For quadrupeds, all the head size comparisons represent substantial increases in correlation over body mass. For the ASC data both head size comparisons change a non-significant correlation with body mass to a strongly significant correlation, with estimated head mass producing the strongest correlation. This discrepancy results from the broad range of relative head sizes found in quadrupedal dinosaurs, which, in contrast to the bipeds, do not have a common body form or relative head size (*e.g.*, head length is 39.3% of body length in *Protoceratops*, but only 1.9% in *Diplodocus*).

For the PSC data, the skull length correlation in quadrupeds is strong and significant, but the head mass correlation does not quite achieve statistical significance at the p = 0.05 level in the standard analysis, although it is below the critical value of 0.754 by only 0.080. This discrepancy is most likely just a sampling artifact. Reliable posterior canal data could not be extracted from the *Protoceratops* specimen used in this study. Within the quadrupedal taxa in this study, *Protoceratops* has the smallest estimated head mass but it has a skull length in the middle of the quadruped range. This means that the absence of *Protoceratops* data from the PSC comparison has a much higher impact on the estimated head mass comparison than the skull length comparison. We hypothesize that, with the inclusion of *Protoceratops* PSC data, the PSC-estimated head mass correlation would be strongly significant. In support of this hypothesis, it is possible to estimate a range of likely sizes for the posterior canal based on the other specimens in the data set. In all specimens measured, the anterior canal is larger than the posterior canal, a situation typical for vertebrates in general [1], [2], [6]. Substituting, therefore, the anterior canal size for the maximum likely posterior canal size of *Protoceratops* we get a correlation coefficient with head mass of 0.712, above the new critical value (n = 8) of 0.707. Similarly, in all but two of the specimens examined in this study (cf. *Gongbusaurus* and *Anchisaurus*) the LSC is smaller than the PSC. When we instead substitute the LSC size for the minimum likely PSC size of *Protoceratops* the correlation coefficient becomes 0.767, also above the new critical value.

Thus, for the whole range of likely sizes for the PSC in *Protoceratops* the correlation with head mass would be significant. The opposite remains true for the correlation with body mass where despite small increases in correlation across the whole range of estimated PSC sizes there is no significance. Inclusion of *Protoceratops* data has a negative effect on the strength of the PSC-skull length correlations which decrease (though remain significant) across the whole range. The non-significant PSC-head mass correlation within quadrupeds could not be compared to the bipedal regression, but it is noteworthy that the significant PSC-head mass regressions which include the theoretical *Protocertops* are significantly and strongly separated from the bipedal regression just as the ASC comparisons were (upper estimate p = 0.002, lower estimate p<0.001). In contrast, the PSC-skull length comparison does not significantly separate bipeds from quadrupeds at the upper limit *Protoceratops* PSC estimate (p = 0.055) and the lower limit estimate only weakly separates the two groups (p = 0.039).

For the LSC data, despite the increase in correlation coefficient with head mass comparison, the correlation for quadrupeds remains 0.272 below the critical value. In contrast, the increase in correlation coefficient with skull length is sufficient to bring this comparison above the level of significance and significantly separate bipeds and quadrupeds. This separation, although significant, is slight and there is substantial overlap of the two groups. This discrepancy is not likely to be a sampling artifact as it was with the PSC. The one taxon missing from this comparison is *Triceratops*, and although *Triceratops* has the second largest estimated head mass in the study it also has the longest measured skull length so the impact of its absence on the two comparisons should be similar. Futhermore, where *Protoceratops* is isolated at the very bottom of the head mass range in this sample, *Triceratops* has an estimated head mass and skull length similar to the related *Agujaceratops* so that, again, the loss of *Triceratops* from these comparisons has a lessened effect.

Thus, there seems to be a pattern in the strength of the discrimination between bipedal and quadrupedal dinosaurs with regards to the head size parameter used. Estimated head mass is much better than skull length at separating the two functional groups by anterior canal area. It is likely, that head mass is also much better than skull length at separating the function groups by posterior canal area. Skull length does not separate bipeds from quadrupeds by posterior canal area, but head mass does when any reasonable estimate of *Protoceratops* PSC area is included in the data. Both of these results conform to the prediction that the midline length of the skull does not represent as robust a proxy of moment of inertia about the rotation axes for the anterior and posterior ducts as does head mass.

The prediction that skull length should, however, be a functionally significant proxy of moment of inertia with regards to the lateral duct is also supported by the fact that skull length separates the functional groups by lateral canal area whereas estimated head mass does not. It is notable, however, that the strength and clarity of this separation is much less than the separations of the vertical canal data by head mass.

This difference in the pattern between the LSC (horizontally oriented) and the ASC and PSC (both vertically oriented) in all dinosaurs may reflect the functional distinction between these systems. When the head is held in normal position, signals from the two vertical semicircular ducts (anterior and posterior), which encode the angular rotations that involve falling, pitch (falling forward or backward) and roll (falling to either side), tend to be used for maintaining balance; signals from the lateral semicircular ducts, which encode the angular rotations that involve turning, tend to be used for navigation [41], [42].

Whereas bipedalism and quadrupedalism present different requirements with respect to balance, there is no expectation that navigation control should differ between these two particular functional groups. It is therefore not surprising to find that this particular division of taxa produces a much weaker functional pattern. It is possible, instead, that lateral duct size is adapted to some other aspect of locomotor behavior which does place demands on navigation such as complexity of environment (e.g., forest versus open plain) or ecological role (e.g., predator versus prey).

Movements of the head are mediated by the neck. Physical properties of the neck (e.g., where flexion occurs most regularly or how much flexion is permitted) may contribute to the effective mass of the region influencing the semicircular ducts. If true, we predict that vertebrates with rigid necks should have larger canals than those with flexible necks given heads of similar size. That is, when a neck is rigid, more of it moves in conjunction with the head. We have shown that the size of the semicircular canals is tied, not to the overall size of the organism, but to the size of the body segment that contains the canals. The mass of this moving head-neck complex is greater than the mass of the head only, which is the isolated moving body segment in an animal with a compliant neck, and, therefore, should be correlated with larger semicircular canals.

There are three examples that support this prediction. Perhaps the most well known is the large semicircular canal size of fish (non-tetrapod vertebrates) [4]. One explanation for the extraordinary size of the canals in fish relates to the viscosity of the endolymph inside the semicircular ducts [43]. With regards to this study, however, fish generally have very large heads for their body size. Whereas many mammals maintain a head mass between 2% and 10% of body mass [44]–[48], the fish *Pagrus major*, for example, has an adult head mass approximately 20% of its body mass [49]. Furthermore, as Mayne pointed out, both the large heads of fish and the lack of neck (*i.e.*, there is a direct connection between the head and shoulder girdle) may explain the extraordinary size of their semicircular ducts [7].

A second example comes from carnivoran mammals. Spoor and Thewissen found, contrary to their predictions, that less agile phocid seals had much larger semicircular canals than more agile and acrobatic otariid seals [13]. Although ottarid seals do have slightly smaller heads for their body size than other mammals [44], this does not explain the oversized canals of phocid seals. Otariid seals primarily locomote using a sub-aqueous flying mode of propulsion and typically have a long, flexible neck [50], [51]. In contrast, phocid seals swim using undulation of the tail, which requires a stiffened anterior body, and as a result, these animals typically hold the head and neck rigid during swimming [50]. This, we argue, results in a more massive effective head and neck functional complex, and in response, the semicircular canals are correspondingly larger.

Lastly, in his study of the natural endocast of the inner ear of *Giraffatitn* (*Brachiosaurus*) *brancai*, Clarke found that the anterior and posterior semicircular canals were much larger than would be expected for its estimated body mass [8]. Sauropod neck posture and, more importantly, flexibility are still widely debated topics [52]–[54]. However, mechanical evidence supports the notion that brachiosaurs had a more stiff neck than many other sauropods [55]. Thus, the greatly enlarged vertical canals in *Giraffatitan* correlate with a more rigid neck. In addition, the same mechanical analysis postulates that *Camarasaurs* had a stiffer neck than *Diplodocus* [55] and, despite having nearly identical head size estimates in this analysis, *Camarasaurus* has an anterior semicircular canal 12% larger in area than *Diplodocus*.

**Figure 5:**
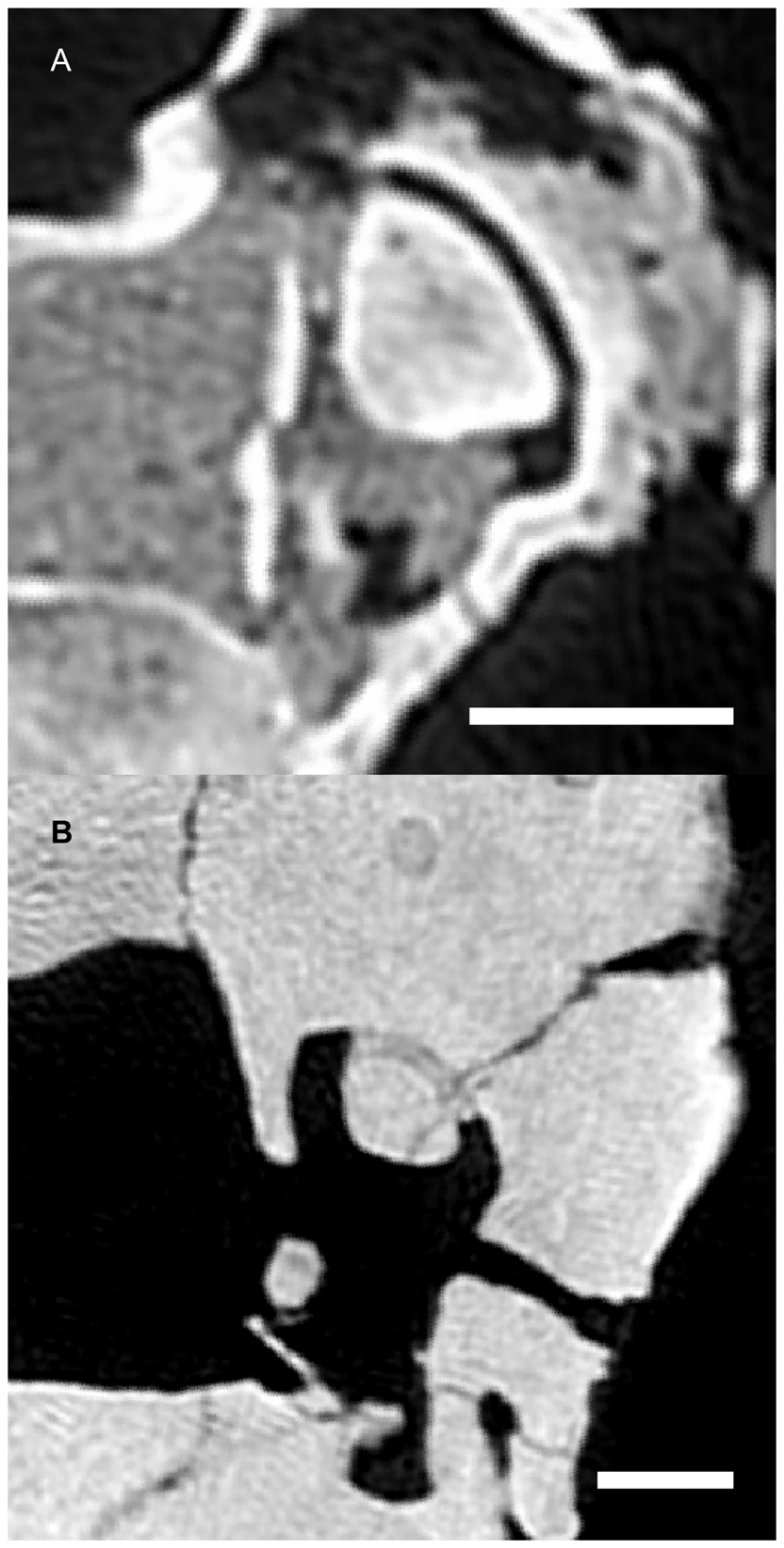
Relative anterior semicircular canal size in bipedal and quadrupedal dinosaurs. Anterior semicircular canal (ASC) in (A) *Psittacosaurus* and (B) *Stegosaurus* scaled to equivalent relative head size using the allometric factor common to the two functional groups. Although the ASC in the small-bodied biped, *Psittacosaurus*, is absolutely smaller than in the larger quadruped, *Stegosaurus*, when the canal systems are scaled as seen here, it is shown that the biped has a markedly larger relative canal. Scale = 10 mm.

The second significant pattern to emerge from this analysis is that the relationship between head mass and semicircular canal size is mediated by the specifics of locomotion. For the anterior canal and likely for the posterior canal as well, bipedal dinosaurs have a larger semicircular canal than quadrupeds of similar head mass (Figure 5). In the ASC, this distinction is represented by a significant difference in the elevation of the two regressions. The elevation shift between the regressions for the PSC would be significant as well, if the quadrupedal regression for those canals was augmented by complete data from *Protoceratops*, as described above. It is not apparent, however, that this general size distinction would be significant for the LSC even if the quadrupedal correlation with head mass achieved significance. While the PSC regression for quadrupedal dinosaurs lacks significance due to data sampling, the LSC data lack significance because of the spread of points around the regression line, including significant overlap with the bipedal taxa. Even when skull length, which is expected to perform better with LSC comparisons, is used the separation between the functional groups, although weakly significant, is not as clear or as diagnostic as the separation with the vertical canals and head mass. Once again, this points to the functional division within the semicircular duct system and the possible separate adaptive function of the LSC.

Our analysis cannot explain the exact nature of the adaptive response of the semicircular ducts to locomotion because it only examines one of several parameters that determine the response profile of the duct, the area enclosed by the duct circuit. Nonetheless, this parameter is informative. The larger the area enclosed by the semicircular duct the greater the force per unit rotation of the duct's fluid on the sensory organ [56]; this would indicate an ability for the system to transduce finer scale movements. Secondly, in taxa with similar duct circuit shapes, this area parameter is approximately proportional to the length of the semicircular duct, which helps determine the slowest movements that will produce a maximum response in the duct system [56]. Therefore, in the absence of the other physiological and morphological parameters of this system in dinosaurs, we hypothesize that this adaptation of larger semicircular duct enclosed areas in bipedal taxa represents an increase in the ability of the vertical semicircular ducts to transduce fine-scale, slow movements. This is consistent with the sensory requirements of a less stable animal, where the smaller the magnitude and slower the detected rotations, the earlier a destabilizing motion is sensed and can be corrected.

The strong distinction between bipedal and quadrupedal dinosaurs when ASC is compared to head mass permits some interpretation of the four contentious taxa in this study (the sauropodomorphs *Anchisaurus* and *Plateosaurus*, the hadrosaurids *Corythosaurus* and *Edmontosaurus*). All are typically described as facultative bipeds, preferentially using a quadrupedal stance during all but the fastest locomotion [57], [58]. The hadrosaurids show an affinity with the quadrupeds in the sample by falling within the range of that group, indicating that their neurosensory systems were adapted to quadrupedal locomotion. Conversely, the two sauropodomorphs exhibit a distinct pattern, with an ASC area that is below the lower limit of the bipeds but above the upper limit of the quadrupeds. The other sauropodomorph in this study, the small bodied *Thecodontosaurus*, is considered fully bipedal [57] and falls within the bipedal range. This may indicate that the two larger-bodied sauropodomorphs regularly relied on both bipedal and quadrupedal locomotion and have a canal system equally adapted to both postures. It is also possible that the somewhat enlarged ASCs in these taxa reflect the plesiomorphic vestibular condition represented by *Thecodontosaurus*, raising the question of the speed with which the vestibular system adapts to locomotor changes.

## Conclusions

This study suggests that use of body mass as a comparison variable for semicircular canal size does not adequately reflect the functional adaptations of the semicircular canal system. Fixed within the head of vertebrates, the semicircular ducts inside the bony canals respond to movements of the head, or to the linked head and neck complex. As a result, the function of the semicircular ducts is tied closely to physical parameters of the head that determine its rotational characteristics, not the physical parameters of the body as a whole. Parameters of head size, therefore, are more theoretically justified choices for comparison variables and are shown to correlate more highly with semicircular canal area than body mass, and in several instances are shown to recover strong and statistically significant correlations where none exist with body mass.

Many previous studies of semicircular canal size using body mass as a comparison variable have found unusual patterns or specific problematic results. This study suggests that these results may well be an artifact of different patterns of head scaling relative to body mass in the taxa being considered, and that these studies should be re-evaluated taking head size into account. For example, attempts to investigate the difference in semicircular canal morphology between bipedal and quadrupedal primates on the basis of body mass have met with mixed results [59], [60]. A study of the evolution of the unique vestibular morphology of modern cetaceans examined some of the more puzzling results with respect to the stiffness of the cervical region in cetaceans, but not with respect to head size [5]. This study suggests that use of head size as the functional comparator might increase the resolution of the comparison and shed more light on puzzling intermediate fossil taxa. Similarly, a study of potential adaptation of the semicircular canals to a subterranean environment in moles found that moles possessed larger semicircular canals than rats of similar body size [9], but did not take into account the numerous specializations of head and neck structure related to burrowing that may result in a significantly more massive head-neck complex which, independent of environment, would result in larger semicircular canals.

The utility of this new method is demonstrated using dinosaurs. It is shown that proper selection of a head size parameter produces better correlations with semicircular canal size than does body mass, and that through the use of an appropriate head size parameter as a comparator, it is possible to resolve functional distinctions not possible through the use of body mass. In this example, it is demonstrated that bipedal dinosaurs are significantly different from quadrupedal dinosaurs on the basis of the size of the vertical semicircular canals relative to head size. Bipedal dinosaurs exhibit significantly larger vertical canals, which we interpret as an adaptation to the less stable bipedal locomotor posture.

Lastly, this method is limited by the quality of the parameters of head size and whether other factors such as the properties of the neck can be taken into account. Single dimension parameters, such as skull length, are unlikely to be equally appropriate for all three canals, but could be used to address questions pertaining to specific canals. More general parameters, such as head mass might be more difficult to obtain reliably, but this study demonstrates that even a rudimentary estimation of head mass performs significantly better than body mass. Furthermore, it is likely that as this work continues better means of estimating head mass or metrics even closer to the functionally relevant moment of inertia can be developed which will improve the ability of this method to resolve functional distinctions within the semicircular canal system.

## Supporting Information

Table S1 **Specimens and measurements used in this study.** All Skull length and Body length values and all Body mass values except for the four taxa listed in Table S2 are taken from the listed references.
(DOCX)

Table S2 **Femur measurements used to calculate body mass estimates in four specimens without reliable literature values.** Diameter and length for *Masiakasaurus knopfleri* are composite values from the six specimens listed in Carano et al. (2002, J Vert Paleo 22:510-534) with femur lengths greater than 170 mm (8).
(DOCX)
