# Supplementary material for: Turning Semicircular Canal Function on Its Head: Dinosaurs and a Novel Vestibular Analysis
Source: PLoS One. 2013 Mar 13;8(3):e58517. doi: 10.1371/journal.pone.0058517 (PMC3596285; doi:10.1371/journal.pone.0058517)
Supplement: Table S1 — Specimens and measurements used in this study. All Skull length and Body length values and all Body mass values except for the four taxa listed in Table S2 are taken from the listed references. (DOCX) [file pone.0058517.s001.docx]

**Table S1: Specimens and measurements used in this study.**

| Taxon | Specimens | Posture | Skull length (m) | Body length (m) | | Body mass (kg) | | Head mass (kg) | | References | | ASC area (mm^2^) | PSC area (mm^2^) | | LSC area (mm^2^) | |  |
| --- | --- | --- | --- | --- | --- | --- | --- | --- | --- | --- | --- | --- | --- | --- | --- | --- | --- |
| *Allosaurus fragilis* | YPM 14554 | B | 0.75 | | 7.4 | | 952.0 | | 96.49 | | [1,2] | - | | - | | 133.43 | |
| *Camptosaurus dispar* | USNM 5473 | B | 0.4 | | 3.5 | | 268.4 | | 30.67 | | [1,3] | 200.27 | | 139.50 | | - | |
| *Citipati osmolskae* | IGM 100/978 | B | 0.16 | | 2.03 | | 138.7 | | 10.93 | | [4] | 109.95 | | 61.68 | | 40.17 | |
| *Dromaeosaurus albertensis* | AMNH 5356 | B | 0.24 | | 2.61 | | 15.0 | | 1.38 | | [5,6] | 94.65 | | 58.97 | | 55.95 | |
| *Gongbusaurus wucaiwanensis* | IVPP 14559 | B | 0.11 | | 0.76 | | 2.0 | | 0.29 | | [4] | 57.96 | | 28.86 | | 51.33 | |
| *Herrerasaurus ischigualastensis* | MCZ 7063 | B | 0.3 | | 5 | | 347.8 | | 20.87 | | [1,7] | 142.16 | | 90.62 | | 68.18 | |
| *Khaan mckennai* | IGM 100/973 | B | 0.12 | | 1.31 | | 13.0 | | 1.19 | | [4] | 67.12 | | 37.00 | | 40.38 | |
| *Masiakasaurus knopfleri* | Unk | B | 0.19 | | 1.8 | | 12.3 | | 1.30 | | [8] | 58.91 | | 32.47 | | 29.59 | |
| *Psittacosaurus mongolensis* | IGM 100/1132 | B | 0.15 | | 1.5 | | 12.1 | | 1.21 | | [1,3] | 78.13 | | 44.18 | | 39.12 | |
| *Struthiomimus altus* | AMNH 5355 | B | 0.25 | | 4.3 | | 175.0 | | 10.17 | | [4,9,10] | 103.35 | | - | | 39.32 | |
| *Tenontosaurus tilletti* | AMNH 3014 | B | 0.64 | | 4.5 | | 242.9 | | 34.55 | | [1,3] | 196.19 | | - | | 68.38 | |
| *Thecodontosaurus antiquus* | YPM 2192 | B | 0.14 | | 2.6 | | 24.6 | | 1.32 | | [1,11] | 73.05 | | - | | - | |
| *Tyrannosaurus rex* | AMNH 5029 | B | 1.21 | | 12 | | 6650.0 | | 670.54 | | [1,2] | 397.36 | | 209.27 | | 184.98 | |
| *Velociraptor mongoliensis* | IGM 100/976 | B | 0.19 | | 2.14 | | 7.0 | | 0.62 | | [1,5] | 75.60 | | 35.07 | | 35.08 | |
| *Agujaceratops mariscalensis* | UTMP 42313-1 | Q | 1.67 | | 5 | | 1658.7 | | 554.01 | | [1,3] | 253.67 | | 184.83 | | 91.99 | |
| *Apatosaurus ajax* | AMNH 1860 | Q | 0.67 | | 21.1 | | 22407.2 | | 711.51 | | [1,3] | 171.36 | | 92.85 | | 72.87 | |
| *Camarasaurus sp.* | CM 11969, AMNH 673 | Q | 0.51 | | 15.4 | | 11652.2 | | 385.88 | | [1,3] | 169.91 | | 100.14 | | 76.34 | |
| *Diplodicus longus* | AMNH 694 | Q | 0.5 | | 25.7 | | 19654.6 | | 382.39 | | [1,12] | 151.60 | | 93.24 | | 44.40 | |
| *Euoplocephalus tutus* | AMNH 5337 | Q | 0.35 | | 6.1 | | 2675.9 | | 153.54 | | [1,3] | 101.40 | | 72.11 | | 45.23 | |
| *Protoceratops andrewsi* | IGM 100/1246 | Q | 0.55 | | 1.4 | | 23.7 | | 9.31 | | [1,3] | 66.24 | | - | | 51.59 | |
| *Stegosaurus armatus* | CM 106, YPM 1853 | Q | 0.39 | | 6.5 | | 2610.6 | | 156.64 | | [1,3] | 110.37 | | 89.30 | | 56.17 | |
| *Triceratops sp.* | LACM 150168 | Q | 1.13 | | 8 | | 4964.0 | | 701.17 | | [1,3] | 278.99 | | 205.74 | | - | |
| *Anchisaurus polyzelus* | YPM 1883 | U | 0.13 | | 2.4 | | 34.0 | | 1.84 | | [13] | 54.34 | | 22.16 | | 32.21 | |
| *Corythosaurus sp.* | AMNH 5433 | U | 0.76 | | 8.1 | | 3078.5 | | 288.85 | | [1,3] | 164.52 | | 131.41 | | 118.67 | |
| *Edmontosaurus sp.* | AMNH 427, YPM 618 | U | 1.11 | | 8.8 | | 3990.8 | | 503.39 | | [1,3] | 179.01 | | 143.48 | | 88.03 | |
| *Plateosaurus longiceps* | AMNH 6810 | U | 0.36 | | 8 | | 2179.0 | | 98.06 | | [3,14] | 162.61 | | - | | 51.09 | |

Table S1: All Skull length and Body length values and all Body mass values except for the four taxa listed in table S2 are taken from the listed references.

1. Seebacher F (2001) A new method to calculate allometric length-mass relationships of dinosaurs. J Vert Paleo 21: 51–60.

2. Chure DJ (1998) On the orbit of theropod dinosaurs. Gaia 15: 233–240.

3. Sereno PC, Zhao X, Brown L, Lin T (2007) New psittacosaurid highlights skull enlargement in horned dinosaurs. Acta Palaeontol Pol 52: 275–284.

4. Christiansen P, Fariña RA (2004) Mass prediction in theropod dinosaurs. Hist Biol 16: 85–92.

5. Therrien F, Henderson DM (2007) My theropod is bigger than yours … or not: estimating body size from skull length in theropods. J Vert Paleo 27: 108–115.

6. Farlow JO, Pianka ER (2002) Body size overlap, habitat partitioning and living space requirements of terrestrial vertebrate predators: implications for the paleoecology of large theropod dinosaurs. Hist Biol 16: 21–40.

7. Sereno PC, Novas FE (1994) The skull and neck of the basal theropod Herrerasaurus ischigualastensis. J Vert Paleo 13: 451–476.

8. Carrano MT, Sampson SD, Forster CA (2002) The osteology of *Masiakasaurus knopfleri*, a small abelisauroid (Dinosauria: Theropoda) from the Late Cretaceous of Madagascar. J Vert Paleo 22: 510–534.

9. Nicholls EL, Russell AP (1981) A new specimen of *Struthiomimus altus* from Alberta, with comments on the classificatory characters of Upper Cretaceous ornithomimids. Can J Earth Sci 18: 518–526.

10. Osborn HF (1917) Skeletal adaptations of *Ornitholestes*, *Struthiomimus*, *Tyrannosaurus*. Bull Am Mus Nat Hist 35: 733–771.

11. Benton MJ, Juul L, Storrs GW, Galton PM (2000) Anatomy and systematics of the prosauropod dinosaur *Thecodontosaurus antiquus* from the Upper Triassic of Southwest England. J Vert Paleo 20: 77–108.

12. Weishampel DB, Dodson P, Osmólska H, editors (2004) The Dinosauria. 2nd ed. University of California Press. 890 p.

13. Christiansen P (1999) On the head size of sauropodomorph dinosaurs: Implications for ecology and physiology. Hist Biol 13: 269–297.

14. Sander PM (1992) The norian Plateosaurus bonebeds of central Europe and their taphonomy. Palaeogeorgr Palaeoclimatol Palaeoecol 93: 255–299.
