# Supplementary material for: Turning Semicircular Canal Function on Its Head: Dinosaurs and a Novel Vestibular Analysis
Source: PLoS One. 2013 Mar 13;8(3):e58517. doi: 10.1371/journal.pone.0058517 (PMC3596285; doi:10.1371/journal.pone.0058517)
Supplement: Table S2 — Femur measurements used to calculate body mass estimates in four specimens without reliable literature values. Diameter and length for Masiakasaurus knopfleri are composite values from the six specimens listed in Carano et al. (2002, J Vert Paleo 22:510-534) with femur lengths greater than 170 mm (8). (DOCX) [file pone.0058517.s002.docx]

**Table S2: Femur measurements used to calculate body mass estimates in four specimens without reliable literature values.**

| Taxon | Specimens | Femur midshaft diameter (mm) | Femur length (mm) |
| --- | --- | --- | --- |
| *Citipati osmolskae* | IGM 100/978 | 43 | 411 |
| *Gongbusaurus wucaiwanensis* | IVPP 14559 | 14 | 103 |
| *Khaan mckennai* | IGM 100/973 | 23 | 190 |
| *Masiakasaurus knopfleri* | Unk | 17 | 195 |

Table S2: Diameter and length for *Masiakasaurus knopfleri* are composite values from the six specimens listed in Carano et al. (2002, J Vert Paleo 22:510-534) with femur lengths greater than 170 mm (8).
